# Multi-Omics Profiling in PGM3 and STAT3 Deficiencies: A Tale of Two Patients

**DOI:** 10.3390/ijms24032406

**Published:** 2023-01-26

**Authors:** Minnie Jacob, Afshan Masood, Anas M. Abdel Rahman

**Affiliations:** 1 Metabolomics Section, Department of Clinical Genomics, Center for Genomics Medicine, King Faisal Specialist Hospital and Research Centre (KFSHRC), Riyadh 11564, Saudi Arabia; 2 Proteomics Resource Unit, Obesity Research Center, College of Medicine, King Saud University, P.O. Box 2925(98), Riyadh 11461, Saudi Arabia; 3 Department of Biochemistry and Molecular Medicine, College of Medicine, Al Faisal University, Riyadh 11533, Saudi Arabia

**Keywords:** signal transducer and activator of transcription 3 (STAT3), phosphoglucomutase 3 (PGM3), dedicator of cytokinesis 8 (DOCK8), hyper IgE syndromes (HIES), multi-omics, metabolomics, proteomics, cytokines profiling

## Abstract

Hyper-IgE Syndrome (HIES) is a heterogeneous group of primary immune-deficiency disorders characterized by elevated levels of IgE, eczema, and recurrent skin and lung infections. HIES that is autosomally dominant in the signal transducer and activator of transcription 3 (STAT3), and autosomal recessive mutations in phosphoglucomutase 3 (PGM3) have been reported in humans. An early diagnosis, based on clinical suspicion and immunological assessments, is challenging. Patients’ metabolomics, proteomics, and cytokine profiles were compared to DOCK 8-deficient and atopic dermatitis patients. The PGM3 metabolomics profile identified significant dysregulation in hypotaurine, hypoxanthine, uridine, and ribothymidine. The eight proteins involved include bifunctional arginine demethylase and lysyl hydroxylase (JMJD1B), type 1 protein phosphatase inhibitor 4 (PPI 4), and platelet factor 4 which aligned with an increased level of the cytokine GCSF. Patients with STAT3 deficiency, on the other hand, showed significant dysregulation in eight metabolites, including an increase in protocatechuic acid, seven proteins including ceruloplasmin, and a plasma protease C1 inhibitor, in addition to cytokine VEGF being dysregulated. Using multi-omics profiling, we identified the dysregulation of endothelial growth factor (EGFR) and tumor necrosis factor (TNF) signaling pathways in PGM3 and STAT3 patients, respectively. Our findings may serve as a stepping stone for larger prospective HIES clinical cohorts to validate their future use as biomarkers.

## 1. Introduction

Hyper-IgE syndrome (HIES) is a group of heterogeneous primary immune-deficiency disorders characterized by elevated levels of IgE, eczema, and recurrent infections as described by Davis et al. in 1966 [1]. Autosomal dominant-negative mutations in the signal transducer and activator of transcription 3 (STAT3), known as Job’s syndrome, were identified in 2007 [2,3]. Recently, two other dominant forms of HIES have been described: an ERBB21P mutation that encodes to protein ERBIN, and CARD11 mutations [4,5], whereas the bi-allelic mutations in the dedicator of cytokinesis 8 (DOCK8) [6] and phosphoglucomutase 3 (PGM3) [7], and interleukin 6 signal transducer (IL6ST) [8] have been identified as the autosomal recessive forms of HIES.

Although the IgE levels are commonly elevated in these conditions, they have different clinical presentations. STAT3 deficiency is a multisystem disease involving the immune system, dentition, skeleton, and connective tissues [9]. Patients with STAT3 signaling defects have fewer food allergies and anaphylaxis than other forms of HIES, which may be due to diminished STAT3 signaling, essential for mast cell mediator-induced vascular permeability [10]. They also present pneumonia due to pneumocystis jiroveci pneumonia (PJP) and mucocutaneous candidiasis on the nails, oropharynx, esophagus, and vaginal mucosa [11]. Hodgkin and non-Hodgkin lymphomas are found at a higher rate in these patients [12]. The recently discovered recessive mutation in PGM3 is due to impaired PGM3 function. It presents elevated IgE levels, atopic dermatitis, bronchiectasis, and scoliosis. Neurocognitive defects and probable hypomyelinations are the distinct characteristics of PGM3 deficiency but not STAT3 or DOCK8. PGM3 is required for combined N and O-glycosylation through catalyzing the isomerization of N-acetylglucosamine-6-phosphate to N-acetyl glucosamine-1-phosphate during the generation of UDP-GlcNAc (Uridine diphosphate N-acetylglucosamine) The UDP-GlcNAc, in turn, is used in downstream glycosylation to make N-glycans and O-glycans through the salvage pathway. The glycosylation of proteins plays an important role in signal transduction and is critical for cell signaling. O-GlcNacylation underlies important cellular mechanisms seen in chronic and neurogenerative diseases. Many oncogenic proteins and tumor suppressor proteins are regulated by O-GlcNAcylation, thereby explaining the pathophysiology of such diseases [13,14].

Increased serum IgE levels are characteristic but not specific to allergic diseases. HIES exhibits an unusual constellation of clinical features. Diagnosis can be confusing and difficult, especially during early childhood, and it is potentially life-threatening, although curable with bone marrow transplantation. Thus, the diagnosis of HIES is critical and should be sought at an early definitive therapy. Severe atopic dermatitis (AD) and HIES share some clinical symptoms, including eczema, eosinophilia, and increased serum IgE levels. AD is a chronic inflammatory disease with remission that commonly occurs in early infancy. Clinical manifestations in patients with AD include food allergy, asthma, and allergic rhinitis, probably in response to impaired innate and adaptive immunity and environmental stimuli [15]. Eczematous skin lesions, pruritus, and food allergies are common overlapping clinical features in HIES and AD.

High throughput “omics” technologies are indispensable for understanding HIES disease’s underlying molecular mechanisms and in-depth pathogenesis as a complex disorder. Multi-omics technologies have been used in several disease’ biomarker discoveries [16]. Significant environmental and clinical disturbances can be monitored at the metabolic level in various pathways crucial for cellular homeostasis [17]. Metabolomics relates to small molecule identification, quantification, and the resulting network interactions representing the individual’s functional genome. The entire qualitative collection of metabolites in a biological sample is called the “metabolome,” which is very dynamic.

Herein, we describe 4 patients with HIES, two of the four with documented mutations in STAT3 and the other two in PGM3. In the current investigation, we combined metabolomics, proteomics, and serum cytokine levels data utilizing a ‘systems biology’ approach to characterize alterations at the biochemical and molecular level in HIES patients using established mass spectrometry and Luminex^®^ xMAP^®^ protocols. The PGM3 and STAT3 distinctive profiles were compared to AD and DOCK8 profiles from previous studies for specificity [18,19,20]. We applied integrative multi-omics methods to these complex diseases to obtain a comprehensive cellular readout offering a holistic view of disease processes. This multi-omics approach can further act as a stepping-stone toward identifying potential biomarkers and early indicators of disease. Identifying these can better connect genotype to phenotype for different forms of HIES and offer effective diagnostic opportunities and molecular targets for treatment.

## 2. Case Presentation

### 2.1. Clinical Characterizations in STAT3 and PGM3 Deficient Patients

The genetic and laboratory characteristics of the study cohorts are represented in Table 1. The mean age of the PGM3 and STAT3 cohorts were 9 ± 7 and 26 ± 2 years, respectively, and that of healthy controls collected from adults was 23 ± 1.03. The PGM3 and STAT3 deficiency group patients presented to the clinics with a history of atopic dermatitis-like skin lesions, eczema, repeated episodes of infections involving the gastrointestinal skin system and viral infections, and multiple food allergies. Patient P2 in the PGM3 deficient group additionally had neutropenia and failure to thrive. On the other hand, patients in the STAT3 group also presented with allergic rhinitis, eosinophilic vasculitis, and episodes of bronchiectasis. The characteristic finding was an increased eosinophil count of 0.1325 ± 0.06 and 0.065 ± 0.018 in the PGM3 and STAT3 deficient patients, respectively. The mean RBCs and WBCs counts in PGM3 patients were 4.8 ± 0.26 (10^12^/L) and 4.2 ± 1.4 (10^9^/L), whereas, in STAT3 patients, they were 4.7 ± 0.02 (10^12^/L) and 5.5 ± 1.9 (10^9^/L), respectively. While eosinophilia was present in all patients, the counts were significantly higher in the PGM3 cohort than in STAT3. On the other hand, no significant difference in neutrophil counts was noted between the two cohorts. Comparatively, the CD4/CD8 ratio in the PGM3 cohort was 1.6 ± 0.7; for one of the STAT3 patients, the ratio was 1.1 (we could not get the ratio for patient P1).

### 2.2. Metabolomics, Proteomics, and Cytokine Profiling in PGM3 and STAT3 Patients

**Figure 1:**
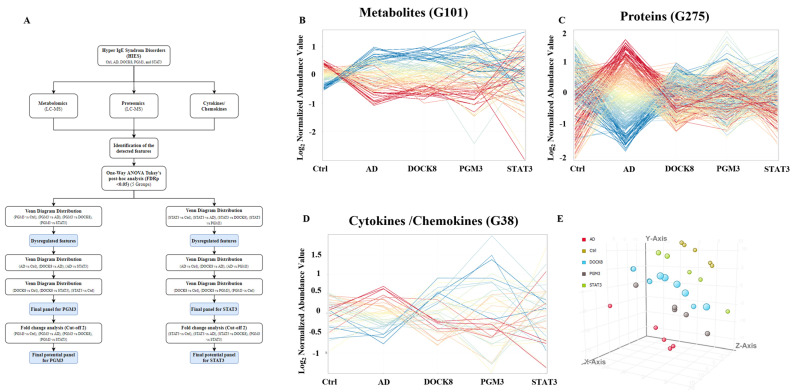
Multi-omics profiles in five study groups and the data analysis pipeline: (**A**) A flow chart showing the data analysis pipeline collected from metabolomics, proteomics, and cytokines platforms. The significant features in each dataset were determined for PGM3 and STAT3 deficiencies, based on One-way ANOVA. Those in common with the other disorders (AD and DOCK) were then excluded using Venn diagram analysis. The final list features were fold change filtered (Cut-off > 2). The expression profile of (**B**) metabolomics (*n* = 101), (**C**) proteomics (*n* = 275), and (**D**) cytokines (*n* = 38) in the five groups. (**E**) A PLS-DA shows a clear separation between the study groups due to the proteomics differential expression.

**Figure 2:**
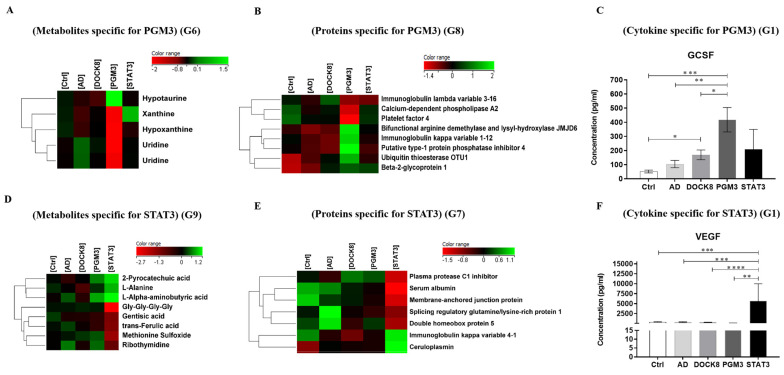
Distinctive Multi-omics profiles for PGM3 and STAT3 deficiencies. Heat maps show specific (**A**) metabolic (n = 6), (**B**) proteins (n = 8), and (**C**) GCSF (Granulocyte-colony stimulating factor) expressions for PGM3 deficiency compared to the other study’s groups. Heat maps show distinctive expression profiles for STAT3 deficiency based on (**D**) metabolomics (n = 8), (**E**) proteomics (n = 7), (**F**) cytokine VEGF (Vascular endothelial growth factor) datasets by entities’ hierarchical clustering for the average normalized data, where the similarity was based on Pearson. Cytokines: Five groups were compared using the One-way ANOVA and Tukey’s tests. * indicates significance. * *p* < 0.05, ** *p* < 0.01, *** *p* < 0.001. Not specified ones are not significant. Abbreviations: Ctrl: Controls, AD: Atopic dermatitis, DOCK8: Dedicator of cytokines8, PGM3: Phosphoglucomutase, 3 STAT3: Signal Transducer Activator of Transcription 3.

Data from five groups (control, PGM3, STAT3, AD, and DOCK8 deficiencies) were collected from these platforms and analyzed, as shown in Figure 1A. The expression profiles for the metabolomics (n = 101), proteomics (n = 275), and cytokines (n = 38) in the study groups are illustrated in Figure 1B–D. To increase the specificity of the identified multi-omics markers, the PGM3 and STAT3 were compared to AD and DOCK8 profiles from our previous studies [19]. One-way ANOVA (Tukey’s post hoc, *p*-value < 0.05) and fold change (FC > 2) analyses showed the significant molecules for each immunodeficiency. Partial least square-discriminant analysis (PLS-DA) demonstrates considerable and distinct separation between the five study group (Figure 1E) profiles. Multi-omics profiles for PGM3 (Figure 2A–C) and STAT3 (Figure 2D–F) were distinctively identified. In PGM3 deficiency, five significantly differentially regulated metabolites revealed dysregulations in the purine (taurine/hypotaurine) and pyrimidine (ribothymidine and uridine) metabolic pathways. (Figure 2A) The proteomics profile showed a significant dysregulation in eight proteins, including an increase in ubiquitin thioesterase, immunoglobulin kappa chains, and protein phosphatase inhibitor 4. At the same time, a decrease was noted in phospholipase A2 and platelet factor 4 expression (Figure 2B). The cytokine profile in PGM3 showed a unique expression of GCSF, as represented in Figure 2C.

Similarly, STAT3 patients revealed significant alterations in eight metabolites, including increased 2-protocatechuic acid, alanine, and aminobutyric acid and decreased methionine sulfoxide and ferulic acid, among others (Figure 2D). The proteomics platform revealed the significant differential regulation of seven proteins (G8), including an increase in ceruloplasmin and the immunoglobulin kappa chains (Figure 2E). The cytokine profile in patients with STAT3 deficiency showed a distinct expression of GCSF (Figure 2F).

### 2.3. Integrated Network Pathway Analysis

We carried out an integrated approach using ingenuity pathway analysis (IPA) software to analyze the ‘omics’ data by incorporating the significantly differential proteins and metabolites in PGM3 (Figure S1) and STAT 3 (Figure S2) immunodeficiencies. The generated network pathways relate to the molecules’ biological interactions in PGM3 deficient patients and are centered on the dysregulation of the endothelial growth factor (EGFR) signaling pathway. The pathway with the highest score was related to cell-cell signaling and interaction, hematological system development, and functional cellular movement (score = 28, Figure S1). On the other hand, the integrated omics analysis for the STAT3-generated network pathway showed the involvement of amino acid metabolism, molecular transport, and small molecule biochemistry with the highest score (score = 30, Figure S2). The differentially regulated proteins and metabolites centered on modulating the actions of the tumor necrosis factor (TNF) signaling pathway (Figure S2).

### 2.4. Materials and Methods

#### 2.4.1. Chemicals

LC-MS grade reagents were purchased from Fisher Scientific (Ottawa, ON), and ^13^C-dansyl chloride was available from the University of Alberta) (http://mcid.chem.ualberta.ca (accessed on 22 July 2021)). For proteomics analysis, the analytical solvents Dithiothreitol (DTT), and Iodoacetamide (IAA) were purchased from Sigma-Aldrich (St. Louis, MS, USA) and RapiGest SF from (Waters, Manchester, UK).

#### 2.4.2. Serum Samples

Serum samples were collected from infants, children, and adults with the clinical and laboratory-confirmed diagnosis of the hereditary DOCK8-deficiency (*n* = 10), AD (*n* = 9), STAT3 (*n* = 2), PGM3 (*n* = 2) and healthy controls (*n* = 33). All recruited subjects attended the allergy/immunology clinics at King Faisal Specialist Hospital and Research Centre (KFSHRC) [17,18]. Patients consented to participate in this study (IRB approval# 2160 015), approved by the Research Ethics Committee at the Office of Research Affairs, KFSHRC. Participating subjects were asked to sign an informed consent form. The physician completed a comprehensive baseline questionnaire, including each patient’s demographics, symptoms, family history, and food allergies. Patients who had received bone marrow transplantation, were enrolled in another clinical study, or were unwilling to provide informed consent were excluded from this study.

#### 2.4.3. Metabolomics Analysis on Chemical Isotope Labeling Liquid Chromatography-Mass Spectrometry (CIL LC-MS)

Serum samples from DOCK8-deficient (*n* = 10), AD (*n* = 9), STAT3 (*n* = 2), PGM3 (*n* = 2), and control samples (*n* = 33) underwent testing using the CIL LC-MS metabolomics platform. The samples were labeled with 12C-dansyl chloride (DnsCl), while a pooled sample was generated by mixing all individual samples and then labeled with 13C-DnsCl. Liquid chromatography with ultraviolet detection (LC-UV) quantitation was performed to determine the total concentration of dansyl-labeled metabolites. Each 12C-labeled sample was mixed with the same molar amount of the 13C-labeled pooled sample and injected into LC-MS. Serum samples were analyzed using a Thermo Fisher Scientific Dionex Ultimate 3000 UHPLC System (Sunnyvale, CA) linked to a Bruker Maxis II quadrupole time-of-flight (Q-TOF) mass spectrometer (Bruker, Billerica, MA, USA), as previously described [17].

#### 2.4.4. Proteomics Analysis on LC-MSE SynaptG2

Similarly, serum samples from DOCK8-deficiency (*n* = 10), AD (*n* = 9), STAT3 (*n* = 2), PGM3 (*n* = 2), and healthy controls (*n* = 33) underwent proteome analysis; 100 µg total protein was subjected to in-solution tryptic digestion, as previously described [19,20]. Proteins were denatured RapiGest reaction quenched with 12 M HCL. Label-free quantitative 1-dimensional Nano Acquity liquid chromatography-tandem mass spectrometry on Synapt G2 (Waters, Manchester, UK) was used to generate expression protein profiles between the sample groups. The instrument settings were optimized as described previously [19,20,21] Data-independent acquisition (MSE)/ion mobility separation was performed and data were acquired over a range of m/z 50–1300 Da using the Mass Lynx programs (version. 4.1, SCN833, Waters, Manchester, UK).

#### 2.4.5. Cytokines/Chemokine Profiling

Patient samples from DOCK8-deficient (*n* = 10), AD (*n* = 9), STAT3 (*n* = 2), PGM3 (*n* = 2), and control samples (*n* = 15) were quantitatively profiled using the MILLIPLEX MAP kit (Millipore) according to the manufacturer’s instructions, where phycoerythrin-conjugated detection was used to form sandwich complexes. The fluorescence data were acquired on the FLEXMAP 3D [18].

## 3. Discussion

In the present study, we described the multi-omics changes in four patients diagnosed with HIES. Genetic mutation analyses identified two patients with a STAT3 mutation and two with a PGM3 mutation. The significant proteins and metabolites in these two data sets were compared to the proteomic, metabolomics, and cytokine profiles of patients with DOCK8 deficiency and AD from our previous studies for specificity [18,22]. Both cohorts’ data were analyzed per the flowchart represented in Figure 1A. Comparing the PGM3 and STAT3 profiles with AD and DOCK8 revealed a distinct separation between the study groups (Figure 1E).

The cases with PGM3 deficiency demonstrated a significant dysregulation of the metabolites of nucleic acid and taurine/ hypotaurine metabolic pathways. The levels of uridine, xanthine, and hypoxanthine significantly decreased, and those of hypotaurine increased. Uridine, the purine nucleotide, is critical to multiple glycosylation pathways for glycan and proteoglycan synthesis via the formation of the sugar nucleotide UDP-GlcNAc. A decreased level indicates a possible disruption in the purine salvage pathways. PGM3 immuno-deficiency altered the nucleotide (purine and pyrimidine) metabolism, as noted in the differential levels of uridine, hypoxanthine, and xanthine. Purines and pyrimidine are building blocks for synthesizing DNA, RNA, metabolic signals, energy transducers, and essentcoenzymes. Studies in animal models have noted that PGM3-mediated sugar nucleotide synthesis is essential for hematopoiesis and overall development [23]. A deficiency in this enzyme and its effects on nucleotide synthesis could account for the failure to thrive, as seen in our patients. Uridine is a nucleotide critical for forming UDP-GlcNAc, which is fundamental for multiple glycosylation pathways for lipids and proteins in different cell types. A decreased level of this metabolite corresponds to glycosylation defects noted in PGM3 immunodeficiency seen more in leucocytes. Based on the Reactome database, the significantly differentially regulated proteins were involved mainly in B-cell production and maturation. Our finding of an increase in immunoglobulin kappa light chain expression in the serum proteomic profile of the patients also supported this. However, the overall lymphocyte count was not altered (Figure 2B, Table 1). Furthermore, the lymphocytes’ defects in glycosylation could also cause a decrease in Platelet factor 4, a heavily glycosylated protein involved in the coagulation pathway. The levels of hypotaurine, a sulfur-containing non-peptidic amino acid, and an antioxidant increased significantly [24]. The multi-omics profile for PGM3 is represented in Figure 2A–C. Another key protein involved in regulating hematopoietic differentiation, JMJD1B, was significantly increased. Animal models using JMJD1B knockout mice displayed defective hematopoiesis, showing moderate anemia and remarkable leukocytosis phenotypes [25]. Among the cytokines, a significant elevation in the levels of GCSF was notable in PGM3-deficient patients compared to the other groups. GCSF is an important hematopoietic growth factor and immune modulator with profound effects on the functional activities of various circulating leukocytes, including T cells, macrophages, neutrophils, endothelial cells, and fibroblasts [26,27]. High levels of G-CSF produce inflammatory cytokines in the skin, leading to greater monocular cell infiltration, especially neutrophils. This could account for the increased skin manifestations seen in these patients. Increased GCSF levels could also serve as a compensatory mechanism to counteract the neutropenia in these patients, similar to P2 in this group [28]. The unique expression of GCSF is associated with bone marrow failure in these patients and is connected with lymphocyte production (Figure 2C). A pathway analysis connecting the significantly dysregulated metabolites, protein, and cytokine identified the network related to cell-cell signaling and interaction, hematological system development and function, and cellular movement (Figure S1). The network mapped to these molecules centers around the epidermal growth factor receptor, is important in innate and humoral immunity, and is crucial in regulating cell proliferation, differentiation, and cellular migration [29].

Two other patients with the clinical phenotype of HIES were identified to have autosomal dominant STAT3 mutations. STAT3 belongs to the STAT family of transcriptional regulators, which are known to play key roles in cell development and differentiation, in addition to cell death. It is also a key regulator of many immunologic pathways transducing signals downstream of many cytokines, including but not limited to IL-6, IL-10, IL-21, GCSF, and the interferon (IFN) family (IFN-γ, IFN-α/β) [30,31]. These patients revealed significant dysregulation in eight metabolites. An upregulation of 2-protocatechuic acid and α-aminobutyric acid, along with a decrease in ribothymidine and methionine sulfoxide, was observed, suggesting the differential regulation of oxidative stress pathways. An upregulation of alanine reflects upon its involvement in T cell activation and memory T cell re-stimulation [32], and could be correlated to the increase in CD4 T cells as noted by the increased CD4/CD8 ratio. An increase in alpha-aminobutyric acid, an isomer of aminobutyric acid, is suggestive of impaired oxidative metabolism, a state of hypercatabolism, and hyperaminoacidemia (Figure 2D) [33]. The proteomics platform in these patients revealed the significant dysregulation of seven proteins (G8), including immunoglobulin kappa variable 4-1, and ceruloplasmin was upregulated. Ceruloplasmin is a positive acute-phase protein with anti- and pro-oxidant activities, reflecting the physiological functions of inflammatory processes. (Figure 2E) An increase in this protein in patients with STAT3 deficiency indicates the presence of an increased inflammatory state. A significant increase in the levels of the cytokine VEGF was observed in patients with STAT3 mutation (Figure 2F). VEGF is an endothelial-cell-specific growth factor that stimulates vasodilation and cell proliferation, increases permeability and migration, promotes endothelial cell survival, and is a potent inducer of vascular permeability [34]. Patients with HIES are known to have vascular abnormalities through VEGF-induced endothelial cell migration involving the STAT3 signaling pathway, which consequently drives vascular remodeling [35,36]. The increase in VEGF is a probable cause for the vasculitis noted in our patients. A pathway analysis in STAT3 deficient patients revealed dysregulation related to amino acid metabolism, oxidative stress, and inflammatory proteins. The differentially regulated molecules all converged around the dysregulation of the TNF signaling pathway (Figure S2). STAT3 signaling is well known to play a crucial role in regulating TNF-α, although with varying effects [37].

Despite having a small cohort, our case report is the first to carry out an integrated multi-omics systems biology approach toward profiling patients with HIES. Our findings showed distinct metabolites, proteins, and cytokines acting in concert to regulate distinct metabolic and signaling pathways that can be targeted for effective treatment and prevention strategies. Future improvements in the application of multi-omics, the generation of larger data sets in more diverse populations, and customized analysis of data types will promote discoveries and shorten the time taken from data generation to publication and translation to clinics. Identifying these mutations will improve the timing and precision of diagnosis and may permit earlier definitive identification.

## 4. Conclusions

An integrated metabolomics analysis identified a distinct molecular pattern in PGM3 and STAT3 patients. The generated integrative data provides a holistic view of a network of interactions and regulatory events underlying disease processes. Our study highlights the importance of using metabolomics, proteomics, and cytokine profiles for the characterization of overlapping but distinct HIES disorders and identifying potential distinguishing biomarkers for early diagnosis. Our findings may form the basis of larger, prospective, externally validated studies in clinical cohorts for their future use as biomarkers.

## Figures and Tables

**Table 1:** Summary of laboratory findings in the patients with PGM3 and STAT3.

Diagnosis	Patient Code	Mutation	Age	Sex	IgE Levels (KU/L)	RBC 10^12^/L	WBC 10^9^/L	Lymphocytes 10^9^/L	Neutrophils 10^9^/L	Eosinophils 10^9^/L	CD4/CD8 Ratio
			(Y)								
**PGM3**	P1	p.A109T	16	M	492	5.15	2.71	2.97	3.05	0.201	0.9
	P2	p.A109T	2	M	2095	4.58	5.7	3.72	1.33	0.064	2.3
**Average ± SEM**			**9 ± 7**	**2/0(M/F)**	**1293.5 ± 801.5**	**4.865 ± 0.26**	**4.205 ± 1.49**	**3.3 ± 0.53**	**2.19 ± 1.21**	**0.1325 ± 0.06**	**1.6 ± 0.7**
**STAT3**	P1	R382Q	28	M	24,330	4.76	3.64	3.81	3.59	0.047	---
	P2	139276: c.2140A > G, p.T714A	24	F	35,650	4.7	7.5	3.13	4.17	0.083	1.1
**Average ± SEM**			**26 ± 2**	**1/1(M/F)**	**29,990 ± 5660**	**4.73 ± 0.02**	**5.57 ± 1.93**	**3.47 ± 0.49**	**3.88 ± 0.41**	**0.065 ± 0.018**	**1.1**

Abbreviations: AD: atopic dermatitis; STAT3: Signal transducer and activator of transcription 3; PGM3: Phosphoglucomutase; F: female; M: male; SEM: standard error of the mean.

## Data Availability

Not applicable.

## Supplementary Materials

The following supporting information can be downloaded at: https://www.mdpi.com/article/10.3390/ijms24032406/s1.
