# Supplementary material for: Multi-Omics Profiling in PGM3 and STAT3 Deficiencies: A Tale of Two Patients
Source: Int J Mol Sci. 2023 Jan 26;24(3):2406. doi: 10.3390/ijms24032406 (PMC9916661; doi:10.3390/ijms24032406)
Supplement: Supplementary file 1 [file ijms-24-02406-s001.zip › ijms-2009555-supplementary.pptx]

## Slide 1
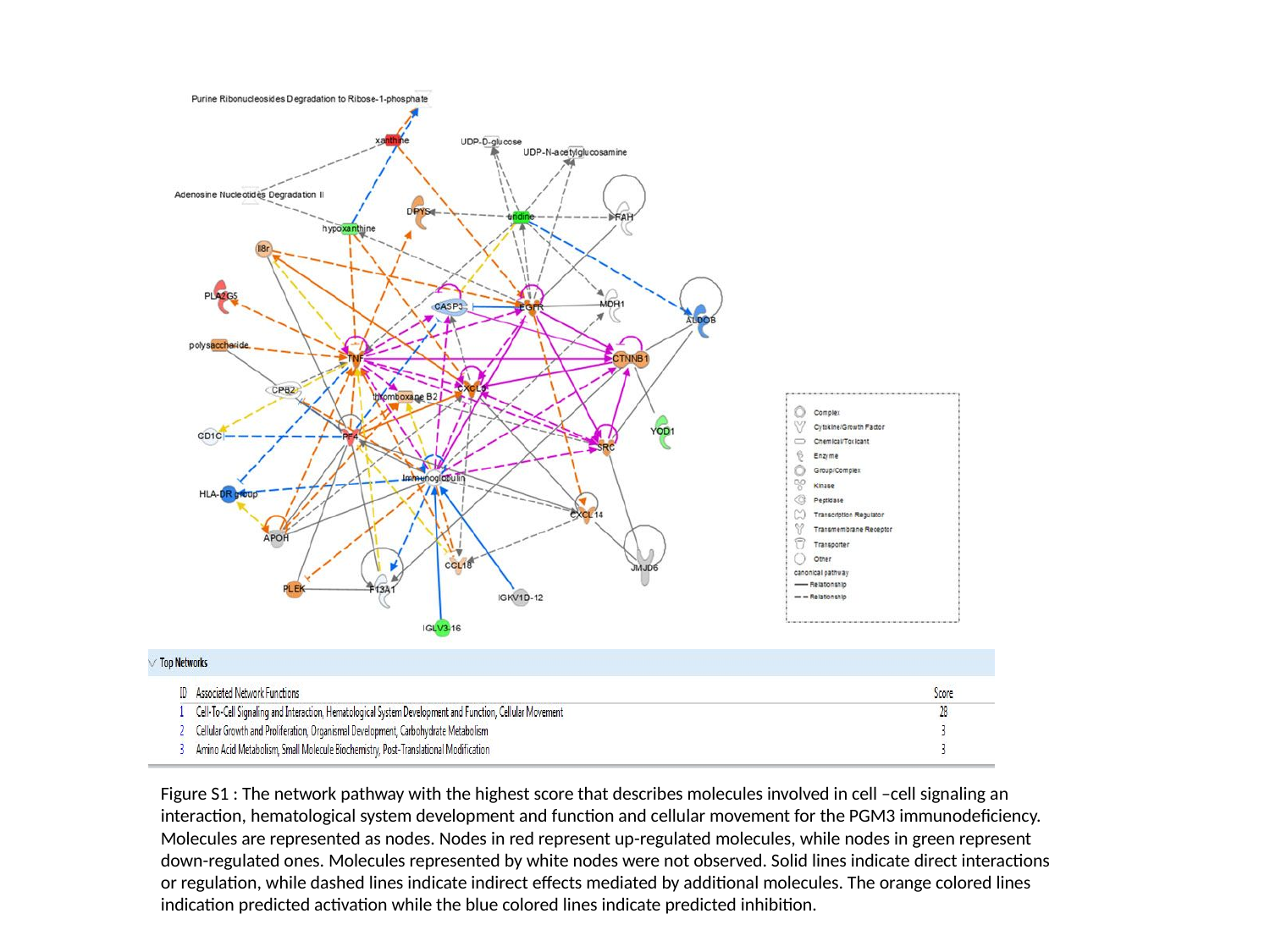

Figure S1 : The network pathway with the highest score that describes molecules involved in cell –cell signaling an interaction, hematological system development and function and cellular movement for the PGM3 immunodeficiency. Molecules are represented as nodes. Nodes in red represent up-regulated molecules, while nodes in green represent down-regulated ones. Molecules represented by white nodes were not observed. Solid lines indicate direct interactions or regulation, while dashed lines indicate indirect effects mediated by additional molecules. The orange colored lines indication predicted activation while the blue colored lines indicate predicted inhibition.

## Slide 2
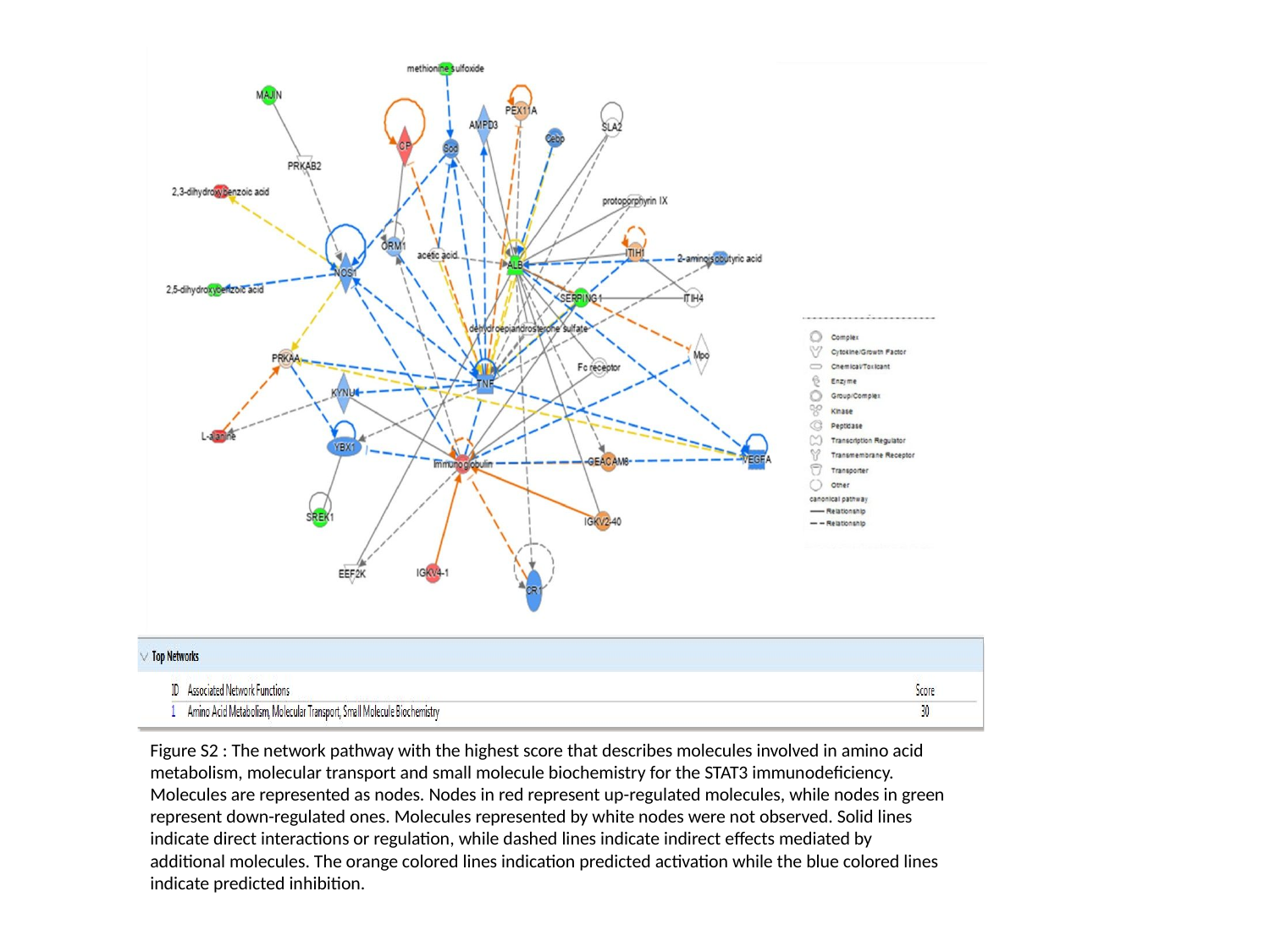

Figure S2 : The network pathway with the highest score that describes molecules involved in amino acid metabolism, molecular transport and small molecule biochemistry for the STAT3 immunodeficiency. Molecules are represented as nodes. Nodes in red represent up-regulated molecules, while nodes in green represent down-regulated ones. Molecules represented by white nodes were not observed. Solid lines indicate direct interactions or regulation, while dashed lines indicate indirect effects mediated by additional molecules. The orange colored lines indication predicted activation while the blue colored lines indicate predicted inhibition.
